# Dual-control of incubation effect for efficiently fabricating surface structures in fused silica

**DOI:** 10.1515/nanoph-2024-0324

**Published:** 2024-08-27

**Authors:** Zhi Wang, Zhikun Xiang, Xiaowei Li, Mengnan Wu, Peng Yi, Chao Zhang, Yihao Yan, Xibiao Li, Xiangyu Zhang, Andong Wang, Lingling Huang

**Affiliations:** Laser Micro/Nano Fabrication Laboratory, School of Mechanical Engineering, Beijing Institute of Technology, No. 5, South Street, Zhongguancun, Beijing 100081, China; Aviation Key Laboratory of Science and Technology on Advanced Corrosion and Protection for Aviation Material, AECC Beijing Institute of Aeronautical Materials, Beijing 100095, China; School of Optics and Photonics, Beijing Institute of Technology, Beijing, 100081, China

**Keywords:** femtosecond laser, etching, efficient, incubation effect, surface structure

## Abstract

Fused silica with surface structures has potential applications in microfluidic, aerospace and other fields. To fabricate structures with high dimensional accuracy and surface quality is of paramount importance. However, it is indeed a challenge to strike a balance between accuracy and efficiency at the same time. Here, a temporally shaped femtosecond laser Bessel-beam-assisted etching method with dual-control of incubation effect is proposed to achieve this balance. Instead of layer-by-layer ablation continuously with Gaussian pulses, silica is modified discretely by double pulse Bessel beam with one single layer. During the modification process, incubation effect is dual-controlled in single shot process and spatial scanning process to generate even modified region efficiently. Then, the modified region is etched to form designed structures such as microholes, grooves, etc. The proposed method exhibits high efficiency for fabrication of surface structures in fused silica.

## 1 Introduction

With the development of society and the progress of science and technology, fabrication of surface structures with high precision is becoming more and more important. As hard material, fused silica is widely used as micro-components in microfluidics [1], [2], [3], micro-optics [4], [5], and sensors [6] and other fields due to its high hardness, high wear resistance, high chemical stability and excellent optical quality. Given the varied requirements across different fields, controlling the morphology accuracy and surface quality of these structures in fused silica is a particularly important task. There are many methods for the manufacturing of surface structures in fused silica, such as machining [7], [8], 3D printing [9], chemical deposition [10], [11] and wet etching [12], [13]. However, it is still difficult to simultaneously balance the processing accuracy and efficiency of hard materials by these methods.

As a non-contact processing method, femtosecond laser is widely used in the processing of surface structures in fused silica with characteristics of high machining accuracy [14], low thermal effect [15] and wide material adaptability [16], [17], [18]. However, limited by the processing mechanism of laser, the machining accuracy and efficiency still remain to be further improved. On the one hand, it will take a lot of time to fabricate structures by ablation with the limited Rayleigh length of Gaussian beam [19]. On the other hand, the continuous surface shape changes and debris accumulation in surface structures ablation process have negative cumulative effects on machining surface quality [20]. Methods such as temporal and spatial shaping of femtosecond laser [21], [22], liquid-assisted ablation [23], [24] have been used to further improve machining accuracy. However, these methods are difficult to balance machining efficiency while improving machining accuracy. Laser-assisted chemical etching is an effectively way to improve the surface quality and manufacturing efficiency [25]. Besides, it can also be used to adjust the structure morphology. However, due to the limited Rayleigh length of Gaussian laser, the improvement in processing efficiency is limited. Besides, the existence of incubation effect makes the fabrication process of laser to be a dynamic process actually which might lead to bad surface quality. As to incubation effect, it can be defined in detail as following, for dielectric materials, laser will induce material modification which involves not only the appearance of defect centres but the change of chemical properties. The modification will result in a decrease in the energy to break atomic bonds, thus reducing the material fluence threshold [26], [27]. When the laser acts on the same area multiple times, the previous modification become seeds, further enhancing subsequent modification and ultimately leading to material removal. This phenomenon has been studied by researchers to fabricate periodic microstructures with high accuracy or remove materials with high efficiency during ablation process [28], [29], [30], [31]. Nevertheless, how to control the degree of modification in the incubation effect to improve the overall uniformity of etching, thus balancing surface quality and manufacturing efficiency still remains a challenge.

This paper proposes dual-control of incubation effect for efficient fabrication of surface structures in fused silica by temporally shaped femtosecond laser Bessel-beam-assisted etching, including laser modification process with dual-control of incubation effect and wet etching process. Using birefringent crystal and axicon, the incident single pulse Gaussian laser beam is formed to double pulse Bessel beam with long focusing depth, which has higher modification degree, modification uniformity and processing efficiency compared with the Gaussian laser with limited Rayleigh length. Therefore, incubation effect is controlled *in situ* in single shot process. Because the Bessel region is fixed, the modification depth is corresponding to the relative position of sample and Bessel region, which enables the rapid and accurate fabrication of single microstructures with different depths. In spatial scanning process, spacing of modification points is strictly regulated to dual-control incubation effect with higher fabrication efficiency and accuracy. Then, these modification points overlap and integrate into a larger scale structure with high quality and high manufacturing efficiency by etching. This method enables surface structures fabrication in fused silica for potential applications in microfluidics and other fields.

## 2 Experimental

### 2.1 Experimental setup and materials

**Figure 1:**
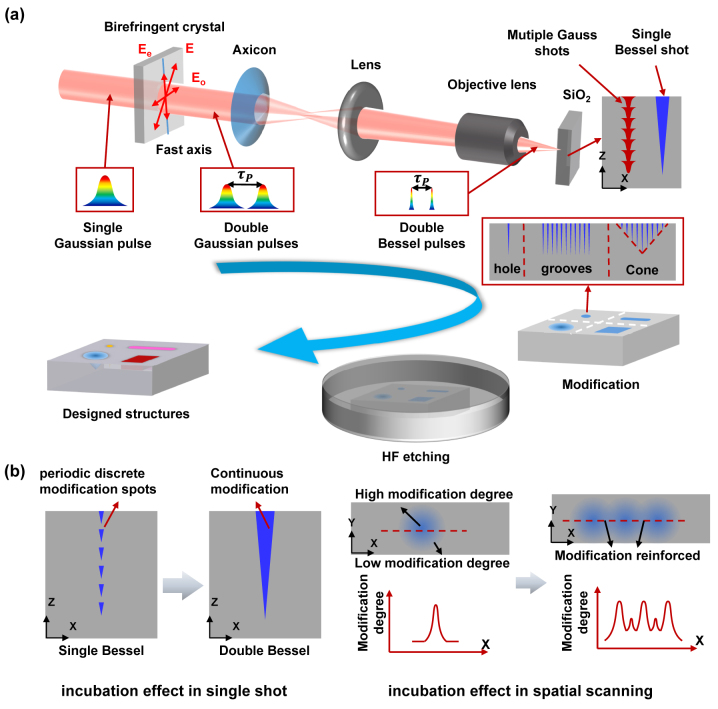
Processing set up and schematic diagram. (a) Schematic of the design and fabrication of designed structures. (b) Schematic for dual-control of incubation effect.

The experimental setup for manufacturing designed structures is illustrated in Figure 1(a). In this study, the femtosecond laser beam (Spitfire Ace-35 F, Spectra-Physics, USA) with a central wavelength of 800 nm, pulse duration of 50 fs and adjustable repetition rate ranging from 1 to 1,000 Hz is used. The single Gaussian pulse is Temporally shaped into a double Gaussian pulse sequence with an adjustable interval of 0 ps–15.42 ps and an energy ratio of 1:1 by BC (birefringent crystal) with different thicknesses (Calcite crystal, Crystik, China). Besides, the double Gaussian pulse is spatially shaped into a double pulse Bessel beam by using an axis cone (Edmund Inc.; base angle *α* = 2°). The relationship between the length of a Bessel region and the base angle is as follows:

(1) $$\beta_{ax}=\arcsin\left(n_{ax}\sin\alpha \right)-\alpha$$

(2) $$Z_{\max}=\frac{w_{0}}{\tan\beta_{ax}}$$

where *β*_*ax*_ is the exit angle of the Bessel beam, *n*_*ax*_ is the refractive index of the axicon, *α* is the base angle of the axicon, and *w*_0_ is the beam radius of the waist of the incident Gaussian beam. Besides, *z*_max_ is the maximum approximate “no diffraction” distance on-axis of the Bessel pulse before it passes through the telescopic system. In this paper, *n*_*ax*_ = 1.45, *α* = 2°, and *w*_0_ = 3 mm.

As Figure 1(a) depicts, the double pulse Bessel beam is then demagnified into dual-pulse micro Bessel beam by a telescope system which consists of a plano-convex lens (Lens; *f* = 150 mm) and a 20× microscope objective (Olympus Inc.; N.A. = 0.45).

(3) $$N=\frac{f_{1}}{f_{2}}$$

(4) $$Z_{\max}^{\prime }=\frac{Z_{\max}}{N^{2}}$$

where *N* is the demagnification ratio, and *f*_1_ is the focal length of the flat-convex lens and *f*_2_ is the equivalent focal length of the objective lens, which are 150 mm and 9 mm, respectively. And $Z_{\max}^{\prime }$ is the length of the Bessel region after the demagnified of the telescope system.

Eventually, the dual-pulse micro Bessel beam is focused into a 1 mm thick, polished fused silica (SiO2, Corning 7,980). After femtosecond laser irradiation, the sample was treated with a hydrogen fluoride (HF) aqueous solution. Particularly, structures with different morphologies can be easily realized by adjusting the modification depth and position of Bessel beam. In addition, due to the long focal depth of Bessel beam, a single shot can achieve high modification depths without the need for multiple modifications with Gaussian beam.

### 2.2 Wet etching

Etching selectivity between the modified and unmodified regions of fused silica is correlated with the concentrations of hydrofluoric acid (HF) [32]. Considering the requirements of etching selectivity and morphology of the designed structures, 20 % HF is selected as the etching agent after laser modification, and the temperature is at room temperature. After etching, the sample is cleaned with water and ethanol for 10 min to ensure no HF residue remained and the sample surface is cleaned.

Schematic for dual-control of incubation effect is shown in Figure 1(b). Firstly, double Bessel pulse train is used to control incubation effect in single shot process to generate continuous modification with high depth in *x*–*z* axes rather than periodic discrete modification spots by single Bessel pulse. Due to the difference of incubation effect, the modification degree is gradually decreased from center to surrounding in single shot process in *x*–*y* axes. Then, to fabricate structures with different morphologies, incubation effect in spatial scanning is studied to control uniformity of modification. While the modification regions overlap each other, the modification degree will be reinforced due to incubation effect. Hence, a local modification enhancement region occurs, causing a fluctuation in the whole uniformity of modification. With appropriate pulse spacing, the fluctuation induced by incubation effect can be controlled well.

### 2.3 Sample characterization

The three-dimensional profile and height of the etched structure were characterized using a laser confocal scanning microscope (LCSM; OLS4100, Olympus). The morphological evolution of the structure with etching time was characterized using an optical transmission microscope (OTM; BX 51, Olympus).

## 3 Results and discussion

### 3.1 Controlling the morphology of microhole structures in single shot

**Figure 2:**
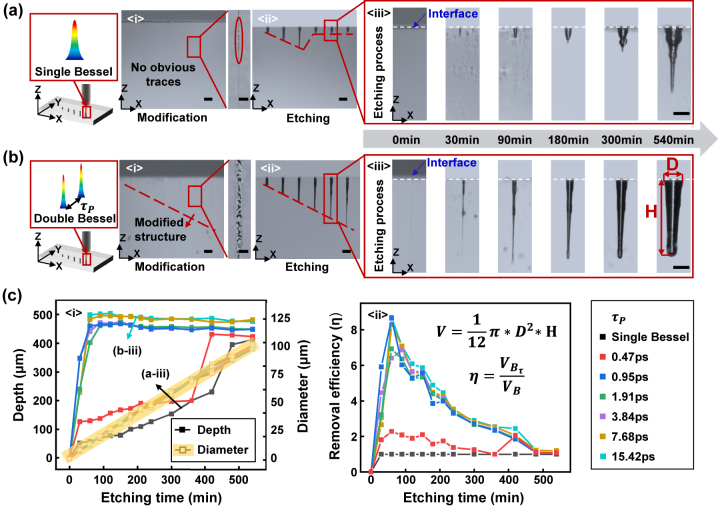
Investigation of incubation effect in single shot. (a) Modification and etching results on microhole structures with gradient depths by single pulse Bessel beam. (b) Modification and etching results on microhole structures with gradient depths by double pulse Bessel beam with pulse delay 15.42 ps. (c) Statistics of etching results of microhole morphology on diameter and depth and removal efficiency with different Bessel beams.

As shown in Figure 2, incubation effect in single shot is investigated by comparing the modification and etching results of single Bessel pulse and double Bessel pulses with different pulse delays. The fused silica is modified by Bessel beam and then etched in HF acid solution to form microhole structures with high depths. In order to control the morphology of the microhole structure better, the etching process under gradient modification depths by Bessel beams with different pulse delays is investigated. Figure 2(a) and (b) depict modified structures with different depths (ranging from 100 μm to 600 μm with an increment of 100 μm) in fused silica, followed by different etching time. Interestingly, visible modified structures are observed in sample processed by double pulse Bessel beams with pulse delay 15.42 ps, while no obvious modified traces are found in samples processed by single pulse Bessel beams. As the enlarged image shows, a line of periodic discrete dots is left with single pulse Bessel. But with the control of incubation effect by double pulse Bessel, the discrete dots shifted to interconnected structures beneficial for diffusion of HF. Subsequent etching process shows irregular morphology by single pulse Bessel beams. In contrast, good etching uniformity and morphology quality are obtained by double pulse Bessel beams with pulse delay 15.42 ps. This difference in etching process can be primarily attributed to the influence of laser modification degree and uniformity that lead to different etching selectivity and uniformity. While the pulse delay is shorter than self-trapped excitons life time, the block of energy deposition of the second subpulse tends to fade away, and the second subpulse may take full advantage of self-trapped excitons contribution, which contributes to the coupling of second subpulse energy into material and the appearance of etching depth enhancement [33].

Obviously, pulse delays and etching time have important impacts on the morphology of surface structures etched. While pulse delay is between 10 and 40 ps, microhole with high aspect ratio and regular morphology can be obtained [33]. Hence, time delays with 0 ps–15.42 ps were studied (more results with different pulse delays can be seen in Figures S1 and S2). Besides, quantitative analysis on the depth and the diameter of microholes was conducted under different etching time, as plotted in Figure 2(c). Statistical analysis with different etching time was performed on the microhole structure with a modification depth of 500 μm. As depicted in Figure 2(c-i). When the pulse delay was greater than 0.47 ps, the structure in depth direction was etched rapidly, reaching the designed modified depth within a short time and remaining stable thereafter. However, when using single Bessel or double Bessel with a pulse delay of 0.47 ps, the depth of microholes increased slowly and irregularly, and the final etching depth was still smaller than the designed modification depth. Notably, the diameter in the radial direction of the microhole structure continued to increase linearly, with an average increment of 13 μm per hour. Figure 2(c-ii) statistically analysed the experimental results of Figure 2(c-i) and calculated the trend of removal volume along with etching time. Due to the similarity between the etched structures and cone, the removed volume (*V*) is calculated according to the formula in Figure 2(c-ii). Besides, the removal efficiency *η* is calculated by the formula in Figure 2(c-ii) where is the removal volume of double Bessel with different pulse delay and is the removal volume of single Bessel. The curve shows that when the etching time is short and the pulse delay is greater than 0.47 ps, there is a higher removal efficiency with the maximum *η* reaches 8.7, which means higher fabrication efficiency. These results are of significant importance for subsequent structures processing and structure morphologies controlling.

### 3.2 Fabrication of groove structures by optimizing incubation effect in spatial scanning

**Figure 3:**
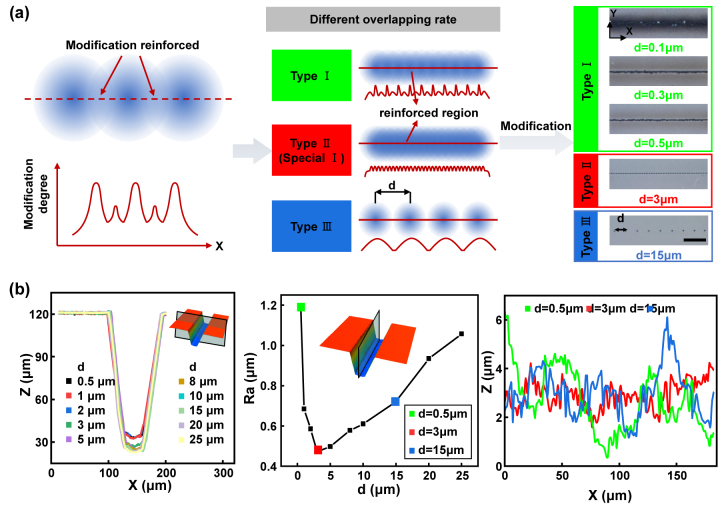
1D groove etching results with different *d*. (a) Schematic diagram of the effect of pulse spacing *d* online etching selectivity. (b) Etching results and surface quality with different *d*.

On the basis of processing microhole structures, we choose a pulse delay of 15.42 ps as the following fabrication parameter. As shown in Figure 3, line scanning was performed to fabricate 1D groove. Comparing to single point modification of microhole, line scanning involves pulse spacing which is defined as *d*. It will influence the modified degree, thereby affecting the surface quality of etched structures as shown in Figure 3(a). For modification with single shot, the modification degree gradually decreases form centre to surroundings. Consequently, while scanning a line, there will be a modification enhanced region with the overlap of modified regions due to incubation effect in spatial scanning. In most cases, this enhancement region may result in uneven modifications, referred to as Type I. However, in certain situations, the degree of modification in the scanning direction is identical, leading to a well-preserved surface quality after etching, known as Type II. Additionally, when the value of “*d*” exceeds the modified area, Type III occurs, where the modified regions do not impact one another. To better illustrate these three types, actual modification results are depicted on the right side of Figure 3(a). Noticeable dark or brown regions appear around the modified points when *d* = 0.1, 0.3, and 0.5 μm. This is partly due to the enhancement modification and partly due to stress accumulation. For *d* = 3 μm, corresponding to Type II, highly uniform modifications are evident. When the *d* = 15 μm, corresponding to Type III, the modified areas do not interact with each other. The corresponding etching results are presented in Figure 3(b). Firstly, the etched structure morphology is characterized and analysed. As *d* increases, the morphology remains consistently well-defined, with a height of 100 μm, matching the depth of modification. Moreover, the surface quality of the structure is assessed through the analysis of bottom line roughness (Ra), revealing that Type II exhibits the lowest Ra value while *d* = 3 μm. When the *d* is below or above 3 μm, the Ra shows an increasing trend. The typical Ra curves for three types (*d* = 0.5, 3, and 15 μm) are displayed on the right side of Figure 3(b). As we can see from the curves, it can be observed that the flatness of *d* = 3 μm is better than the others. This can be seen as an increase in the overall uniformity of modification during the incubation process. In conventional modification enhancement, such as *d* = 0.5 μm, a waveform with alternating main and sub peaks will be formed, resulting in decreased etching uniformity and higher surface roughness. As the pulse spacing approaches a specific value (*d* = 3 μm in this article), the main peak and sub peak become closer and even the same. At this point, the overall modification uniformity is the highest, which inducing a smooth structure with lowest surface roughness.

**Figure 4:**
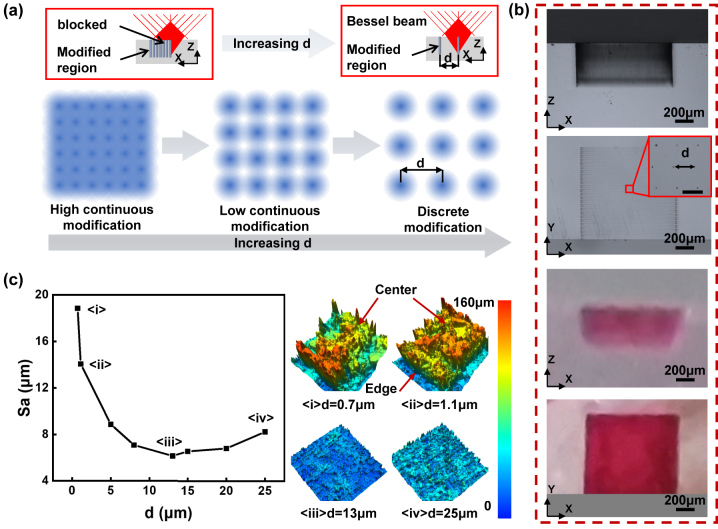
2D groove etching results with different *d*. (a) Schematic diagram of the effect of pulse spacing *d* on the selectivity of planar etching. (b) Modification and etching results with *d* = 20 μm, scale bar: 20 μm (the inset figure). (c) Etching results and surface quality with different *d*.

**Figure 5:**
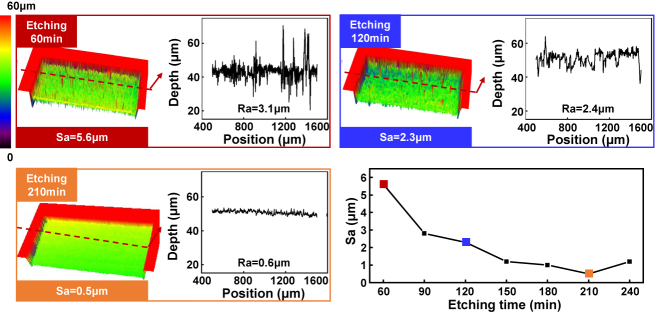
The effect of etching time on the roughness of the bottom surface.

When the 1D groove transforms into 2D groove, the effect of *d* on modification and etching differs. As shown in Figure 4(a), when *d* is small, the enhancement of modification will have a cumulative effect in both the *x* and *y* directions. However, as *d* increases, the mutual influence between modified regions gradually weakens. This is similar to the modification of 1D groove. However, the difference lies in the fact that due to the refractive index changes caused by the modified regions, this may have adverse effects on subsequent laser modification, especially when *d* is small. Parts of the Bessel beams are absorbed multiple times by modified regions by incubation effect. Thus, Bessel beam is blocked during propagation, leading to a decrease modification degree in the region we actually want to modify. As *d* increases, the influence between modified regions gradually weakens and eventually disappears. Figure 4(b) presents the modification and etching results with *d* = 20 μm. It can be seen that the 500 μm modification depth has been completely etched with smooth sidewalls for the 2D groove, but the bottom surface has higher roughness. This can be further reduced by controlling the etching time, which will be studied in Figure 5. The morphology accuracy and surface quality of 2D grooves with various *d* are then studied, as shown in Figure 4(c). The surface roughness (Sa) is regarded as an evaluation criterion for surface quality with different *d*. It can be observed that as *d* increases, the Sa decreases first and then increases, reaching its minimum at *d* = 13 μm. The related confocal images of etched surfaces for *d* = 0.7, 1.1, 13, and 25 μm are shown on the right side of Figure 4(c) (more results with different pulse distances can be seen in Figures S3 and S4). It can be seen that when *d* = 0.7 and 1.1 μm, the etching is incomplete. The edge positions reach the depth set while the central parts present a poor etching state. It can be attributed to the incubation effect reinforced in nearby modified region described before. When *d* is 13 μm and 25 μm, the entire modified area is completely etched but with different Sa.

### 3.3 Investigation of relationship between surface quality and etching time

In addition to the pulse interval *d*, the etching time also has a significant impact on the structure morphology accuracy and surface quality. On the one hand, as the etching time increases, the size of the 2D grooves in the *x*–*y* direction increases at a rate of 13 μm/h, as observed in the experimental results shown in Figure 2. Therefore, in practical machining processes, it is necessary to reserve a certain machining allowance based on the etching time. On the other hand, as the etching time increases, the surface quality will get better due to smoothening effect in low selectivity etching process. Hence, this study further investigates the influence of the etching time on the surface quality. As shown in the Figure 5, due to the differences in local etching rates during the etching process, peak-valleys will appear which induce to a surface structure with high roughness at the beginning. However, with the increase of etching time, the bottom surface gradually becomes smoother and the roughness decreases. When the etching time *t* = 210 min, the Sa reaches the minimum 0.5 μm. However, Sa cannot be reduced infinitely due to the reaction product deposited on surface of fused silica [34].

### 3.4 Fabrication of multiscale structures

**Figure 6:**
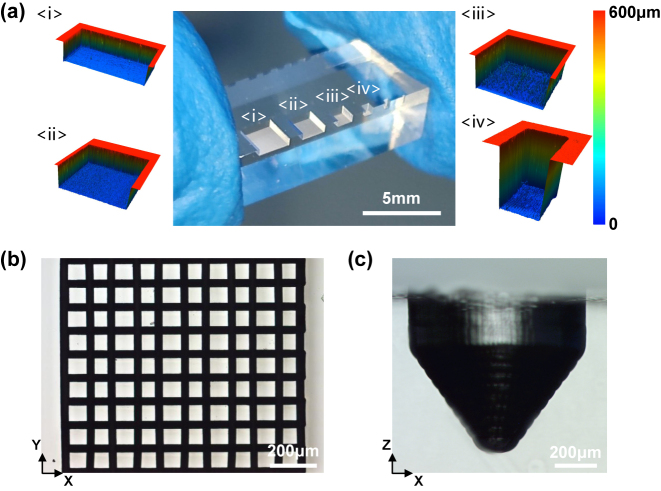
Structures with different morphologies after etching.

The grooves can be fabricated with high quality by the double pulse Bessel beam modification assisted with HF wet etching as depicted in Figures 3 and 4. The pulse spacing and etching time both affect the accuracy of structures. Structures with different morphology can also be fabricated and applied to various field. With the flexible processing ability of femtosecond laser, designable patterns can be fabricated with high accuracy and efficiency. Grooves with different size are fabricated as shown in Figure 6(a). Besides, a microcolumns array and a millimetre scale micro-cone are presented in Figure 6(b) and (c). The morphology evolution of micro-cone structure with etching time can be seen in Figure S5.

## 4 Conclusions

In summary, a technique for fabricating high precision structures with different morphologies with the dual-control of incubation effect by temporally shaped femtosecond laser Bessel-beam-assisted etching is proposed. The fused silica is processed by single plane scanning with double Bessel pulses to generate modified areas of different morphologies, which formed precision structural parts after HF etching. For the structural parts manufactured by this technology, the processing efficiency is increased, and it has an extremely high energy utilization rate and good etching uniformity and accuracy. Moreover, by extending the etching time, the minimum roughness of the structural parts is less than 1 μm. It provides a new concept for balancing efficiency and accuracy in the processing of surface structures.

## Supplementary Material

Supplementary Material Details
