# Supplementary material for: Dual-control of incubation effect for efficiently fabricating surface structures in fused silica
Source: Nanophotonics. 2024 Aug 27;13(21):4057–65. doi: 10.1515/nanoph-2024-0324 (PMC11501070; doi:10.1515/nanoph-2024-0324)
Supplement: Supplementary file 1 — Supplementary Material Details [file j_nanoph-2024-0324_suppl_001.pdf]

Supporting Information

Dual-control of incubation effect for efficient fabrication in fused silica

Zhi Wang, Zhikun Xiang, Xiaowei Li*, Mengnan Wu, Peng Yi, Chao Zhang, Yihao Yan, Xibiao Li, Xiangyu Zhang, Andong Wang, Lingling Huang

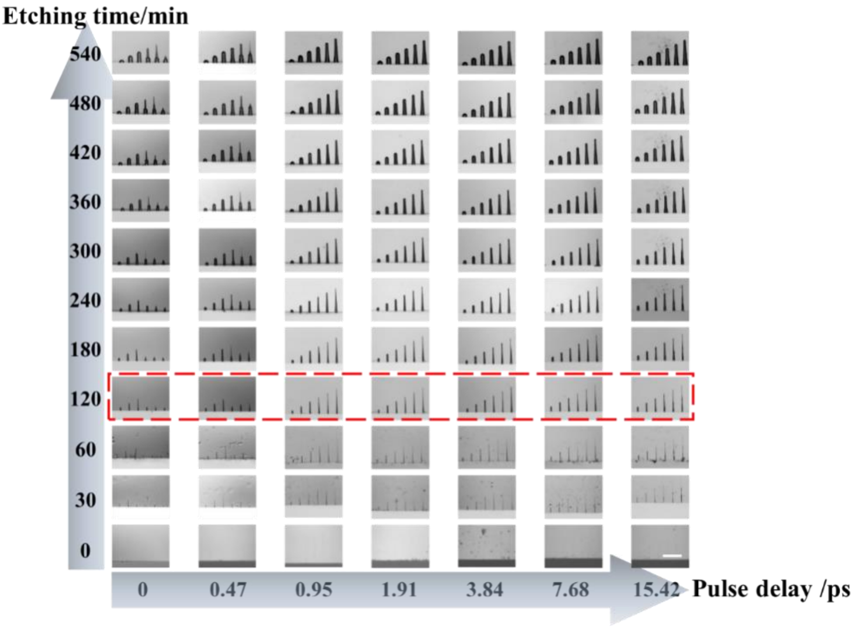

Figure S1. The morphological evolution of gradient height micro-cones with different pulse delays over etching time. (scale bar：400μm)

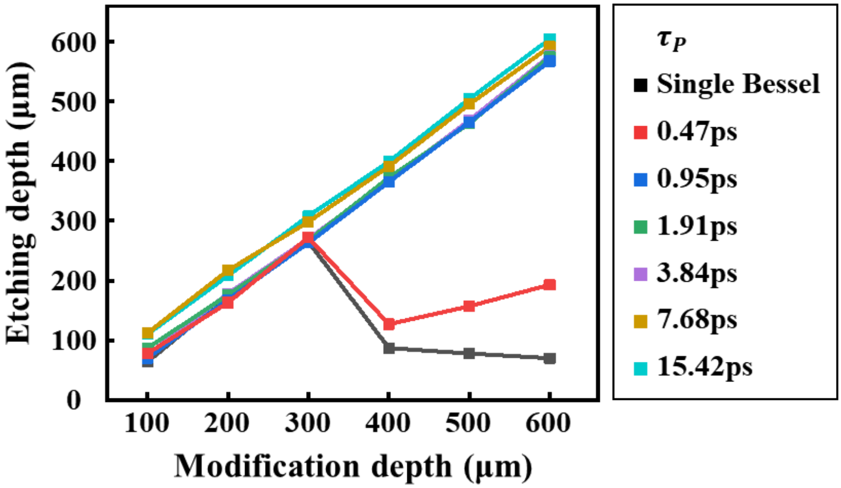

Figure S2. Statistical curves between etching depths and modification depths of gradient height micro-cones with different pulse delays while etching 120 min. (corresponding to the red dashed box in Figure S1)

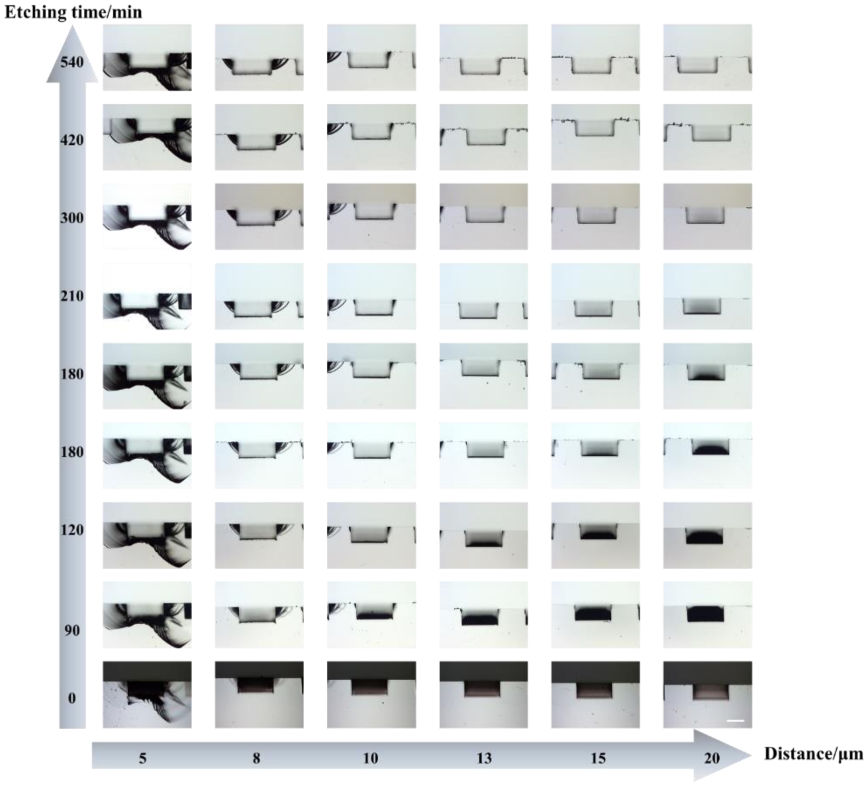

Figure S3. Morphological evolution of 2D grooves with different pulse distance over etching time. (scale bar：500μm)

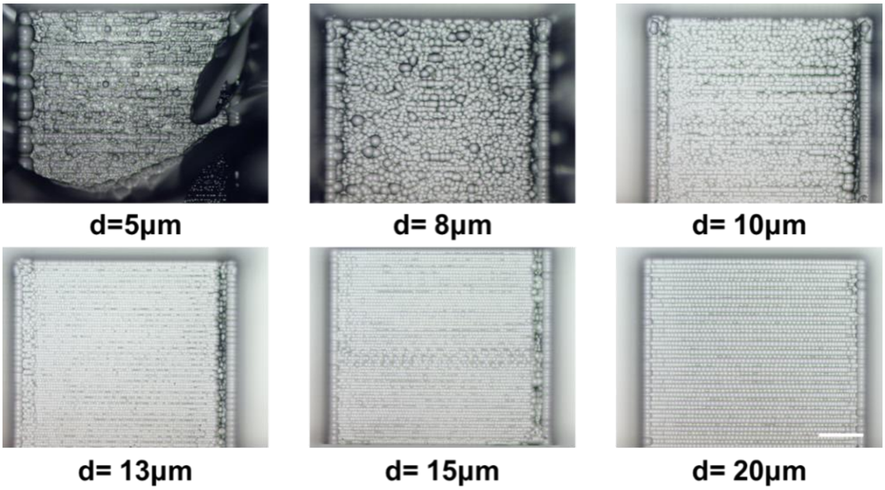

Figure S4. The bottom morphologies of 2D grooves with different pulse distance while etching 480 min. (scale bar：200μm)

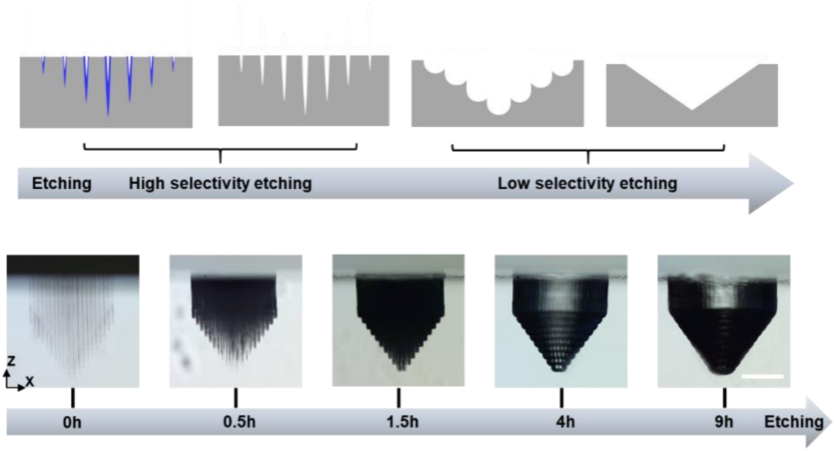

Figure S5. The schematic and OTM images of millimetre scale micro-cone structure morphology evolution with etching time. (scale bar：200μm）
